# Variation in outcomes and practice patterns among patients with localized pancreatic cancer: the impact of the pancreatic cancer multidisciplinary clinic

**DOI:** 10.3389/fonc.2024.1427775

**Published:** 2024-07-11

**Authors:** Priya Pathak, Amy Hacker-Prietz, Joseph M. Herman, Lei Zheng, Jin He, Amol K. Narang

**Affiliations:** ^1^ Department of Radiation Oncology, Johns Hopkins School of Medicine, Baltimore, MD, United States; ^2^ Department of Radiation Oncology, Northwell Health, New Hyde Park, NY, United States; ^3^ Department of Medical Oncology, Johns Hopkins School of Medicine, Baltimore, MD, United States; ^4^ Department of Surgical Oncology, Johns Hopkins School of Medicine, Baltimore, MD, United States

**Keywords:** multi-disciplinary clinic, neoadjuvant therapy, pancreatic cancer, clinical trial enrollment, genetic testing, treatment adherence

## Abstract

**Introduction:**

Patients with localized pancreatic adenocarcinoma (PDAC) benefit from multi-modality therapy. Whether care patterns and oncologic outcomes vary if a patient was seen through a pancreatic multi-disciplinary clinic (PMDC) versus only individual specialty clinics is unclear.

**Methods:**

Using institutional Pancreatic Cancer Registry, we identified patients with localized PDAC from 2019- 2022 who eventually underwent resection. It was our standard practice for borderline resectable (BRPC) patients to undergo ≤4 months of neoadjuvant chemotherapy, ± radiation, followed by exploration, while locally advanced (LAPC) patients were treated with 4-6 months of chemotherapy, followed by radiation and potential exploration. Descriptive and multivariable analyses (MVA) were performed to examine the association between clinic type (PMDC vs individual specialty clinics i.e. surgical oncology, medical oncology, or radiation oncology) and study outcomes.

**Results:**

A total of 416 patients met inclusion criteria. Of these, 267 (64.2%) had PMDC visits. PMDC group received radiation therapy more commonly (53.9% versus 27.5%, p=0.001), as compared to individual specialty clinic group. Completion of neoadjuvant treatment (NAT) was far more frequent in patients seen through PMDC compared to patients seen through individual specialty clinics (69.3% vs 48.9%). On MVA, PMDC group was significantly associated with receipt of NAT per institutional standards (adjusted OR 2.23, 95% CI 1.46-7.07, p=0.006). Moreover, the average treatment effect of PMDC on progression-free survival (PFS) was 4.45 (95CI: 0.87-8.03) months. No significant association between overall survival (OS) and clinic type was observed.

**Discussion:**

Provision of care through PMDC was associated with significantly higher odds of completing NAT per institutional standards as compared to individual specialty clinics, which possibly translated into improved PFS. The development of multidisciplinary clinics for management of pancreatic cancer should be incentivized, and any barriers to such development should be addressed.

## 1 Introduction

Pancreatic cancer is estimated to be the third leading cause of all cancer deaths in 2023 and is projected to become the second most leading cause of cancer deaths by 2024, surpassing colon and rectal cancer (1). With an overall survival (OS) of 12%, it has the lowest survival rate among all cancers.

Given that the standards of care for localized pancreatic ductal adenocarcinoma (PDAC) remain controversial, there is consensus that treatment options for pancreatic cancer require a multi-modality approach (2). Indeed, as an example, during the last decade there has been growing support for neoadjuvant treatment (NAT) for resectable disease (3–5), but a recent randomized controlled trial that compared neoadjuvant versus upfront resection for resectable PDAC showed no survival differences among the two groups. Similarly, in the borderline resectable setting, while the role of neoadjuvant chemotherapy has been established as a standard of care, the optimal duration of chemotherapy prior to local therapy is unclear, as is the role of pre-operative radiation therapy (6, 7). Furthermore, the duration of chemotherapy and role of radiation therapy has not been established for locally advanced pancreatic cancer (LAPC) (8–10).

Due to the lack of consensus in treatment recommendations, multidisciplinary cancer clinics (MDC) are increasingly being utilized at high-volume cancer centers (2, 11). Previous studies have found that MDC are associated with improvement in multiple outcomes, including wait times, clinical trial accrual, and disparities in care (12, 13). Our primary goal was to examine whether evaluation through a pancreatic MDC (PMDC) was associated with improved adherence to treatment recommendations based on institutional standards as compared to evaluation in a specialty-specific clinic.

## 2 Methods

### 2.1 Data source and study population

This is a retrospective observational cohort study of an institutional registry to identify patients undergoing definitive surgical resection between 2019 and 2022 and had a primary tumor with histological diagnosis of pancreas ductal adenocarcinoma or adenosquamous carcinoma. Patient demographic, clinical, operative, pathologic, and outcomes data were extracted from the tumor registry. Patients who were above 18 years old were included in the cohort. Patients who had a preoperative diagnosis of metastatic disease, or had aborted or palliative surgeries, were excluded. The project was deemed exempt by the Johns Hopkins School of Medicine Institutional Review Board.

### 2.2 Neoadjuvant therapy

During the study period, it was standard practice for borderline resectable (BRPC) patients to undergo at least 4 months of neoadjuvant chemotherapy, with or without pre-operative radiation, before transitioning to exploration, while locally advanced (LAPC) patients were treated with 4-6 months of chemotherapy, followed by radiation, followed by potential exploration. In the study, we scored patients as having completed NAT per institutional standards if those with BRPC received 4 months of chemotherapy with or without radiation prior to surgery and those with LAPC received at least 4 months of chemotherapy and radiation prior to the surgery. With respect to radiation therapy, patients could receive either hypofractionated stereotactic body radiation therapy or fractionated radiation, the latter often with concurrent chemotherapy. Due to lack of consensus on the oncologic benefit of NAT for patients with resectable tumors, standard institutional practice was to discuss the relative merits of a surgery-first approach vs a neoadjuvant therapy-first approach and make a joint decision with patients. Therefore, resectable cases were excluded from the analysis for the outcome of completion of NAT per institutional standards. All patients included in the study underwent surgical resection at Johns Hopkins but may have received chemotherapy and/or radiation at an outside facility.

### 2.3 Clinic type

The PMDC at Johns Hopkins Hospital (JHH) was established in 2006 and provides a comprehensive multimodality consultation with surgical, medical and/or radiation oncology, along with provision of ancillary services, in a single visit (14). The clinic is conducted once per week and includes primarily newly diagnosed patients, but also patients returning for restaging or coming for a second opinion. The study cohort was divided into those seen through PMDC at any point during their pre-operative care versus those who were strictly seen through individual specialty clinics at JHH (i.e., surgical oncology, medical oncology, or radiation oncology clinics). Of note, while most patients with localized pancreatic cancer are triaged through our PMDC, some patients or referring physicians request visits with specific providers, which generally is the reason for being seen outside of our PMDC. Notably, we do not have a uniform MDC referral practice pattern for patients who decide to go to a specialty specific clinic first. It is under the discretion of the provider to refer the patient to MDC following the patient’s visit if they choose. Patients who were categorized as part of the PMDC group in the study had at least one PMDC visit at JHH. Also of note, after a patient is seen in PMDC, they are generally offered follow-up in PMDC every two months for restaging and evaluation of the course of the disease.

### 2.4 Study variables and outcomes

The primary outcome of the study was completion of neoadjuvant therapy per institutional standards. Various covariates included in the study were age at diagnosis, sex, race, surgical stage, performance status, comorbidities such as hypertension, diabetes, and hyperlipidemia, and history of another cancer besides PDAC. In addition, family history of pancreatic cancer and other cancers were included. Performance status was determined using the Eastern Cooperative Oncology Group (ECOG) scale (15). Patients with ECOG 0-1 were categorized as having a *good* performance status while patients with ECOG ≥2 were defined as having a *poor* performance status in the multivariable analyses. The secondary outcomes of the study were defined as follows:

- Clinical trial enrollment: defined as patient enrollment in any therapeutic study at JHH prior to surgery.
- Genetic testing prior to surgery: defined as completion and reporting of either germline or somatic testing prior to surgery.
- Pathologic end points of interest: Neoadjuvant tumor response, lymph node status during surgery, and surgical margin status were considered. Details along with their definitions used in the study were described in **Supplementary 1**.
- Progression-free survival, defined in months from the date of surgery to the first recorded date of recurrence. All sites of recurrence including local and distant were considered in the calculation of PFS.
- Overall survival: defined in months from the date of surgery to the date of death. Only patients who died during the study period were included in the calculation while patients who were alive were censored at time of last follow-up.

### 2.5 Statistical analysis

Descriptive statistics were used to report various demographic and clinicopathological characteristics such as frequencies (percent) and median [Interquartile range (IQR)]. The Mann-Whitney U test and chi squared tests were used to compare continuous and categorical variables, respectively. Multivariable logistic regression was performed to find an association between clinic type and various primary and secondary outcomes after controlling for the following covariates: age, sex, race, performance status, stage, NAT, duration of chemotherapy, and baseline CA19-9 level. With clinic types handled as treatment groups, the survival treatment estimates for OS and PFS were calculated using cox proportion hazard model. Additionally, inverse probability weighting was used to estimate treatment effects of PMDC as potential-outcome means (POMs) and average treatment effects (ATEs) using observational data (16). An α of 0.05 was set for significance. All analyses were performed using Stata 18.

## 3 Results

### 3.1 Demographics and clinical characteristics

A total of 416 patients met the inclusion criteria, of which, 64.2% (n=267) were seen in the PMDC at least once during their treatment. Of the patients in the PMDC group, 25.5% (n=68) had multiple PMDC visits. **Table 1** summarizes demographic, clinical, and treatment characteristics of the patient cohort, stratified by clinic visit type. Of interest, the cohort of patients seen in PMDC contained a higher proportion of white patients (n=215, 80.5% vs n=106, 71.1%) and a lower proportion of Black patients (n=16, 6.0% vs. n=18, 12.1%) as compared to the cohort of patients seen in individual specialty clinics (overall p=0.047). There was also a difference in the distribution of stage of disease between the patients seen in PMDC versus those seen only through individual specialty clinics, with the PMDC cohort containing a higher proportion of LAPC patients (n=89, 33.3% vs. n=27, 18.1%) (overall p=0.004). Other pertinent details are summarized in **Table 1**.

**Table 1:** Demographic, clinical, and treatment characteristics of patients receiving neoadjuvant therapy.

	Total	Individual Specialty Clinic (n=149, 34.8%)	PMDC (n=267, 64.2%)	p-value
Age, Median (IQR) in years	67 (60-73)	68 (59-74)	66 (60-73)	0.569
Sex				0.096
Males Females	207 (49.8%) 209 (50.2%)	66 (45.3%) 83 (55.7%)	141 (53.2%) 126 (47.2%)	
Race				0.047
Whites	321 (77.2%)	106 (71.1%)	215 (80.5%)	
Blacks	34 (8.2%)	18 (12.1%)	16 (6.0%)	
Others	61 (14.7%)	25 (16.8%)	36 (13.5%)	
Previous History of Cancers other than PDAC	87 (20.9%)	24 (16.1%)	63 (23.6%)	0.072
Family history of PDAC	69 (16.6%)	22 (14.8%)	47 (17.6%)	0.456
Family history of other cancers	303 (72.8%)	92 (61.7%)	211 (79.0%)	<0.001
Treatment Naïve CA 19-9 in U/ml	192 (48-627)	248 (76-762)	162 (40-586)	0.126
ECOG				<0.001
0-1	356 (85.6%)	105 (70.5%)	251 (94.0%)	
2-3	15 (3.0%)	7 (4.7%)	8 (3.0%)	
Unknown	45 (10.8%)	37 (24.8%)	8 (3.0%)	
Stages of Disease				0.004
Resectable	147 (35.3%)	59 (39.6%)	88 (33.0%)	
Borderline Resectable	153 (36.8%)	63 (42.3%)	90 (33.7%)	
Locally Advanced	116 (27.8%)	27 (18.1%)	89 (33.3%)	
Total months of Chemotherapy, Median (IQR)	4 (3-5)	3 (2-4)	4 (3-5)	0.027
First agent				0.018
5-FU based	322 (79.5%)	106 (72.1%)	216 (83.7%)	
Gem-based	78 (19.3%)	38 (25.8%)	40 (15.5%)	
Immunotherapy	5 (1.2%)	3 (2.0%)	2 (0.8%)	
Switch of Chemotherapy	52 (12.5%)	17 (11.4%)	35 (13.2%)	0.656
Neoadjuvant Radiation	185 (44.5%)	41 (27.5%)	144 (53.9%)	0.001
SBRT	136 (73.5%)	22 (53.6%)	114 (79.2%)	0.005
Fractionated Radiation	49 (26.5%)	19 (46.3%)	30 (20.8%)	

IQR, Interquartile range, PDAC, Pancreatic ductal adenocarcinoma; ECOG, Eastern Cooperative Oncology Group. PMDC: Pancreas Multidisciplinary Clinic; SBRT: Stereotactic Body Radiotherapy.

### 3.2 Treatment characteristics

**Figure 1:**
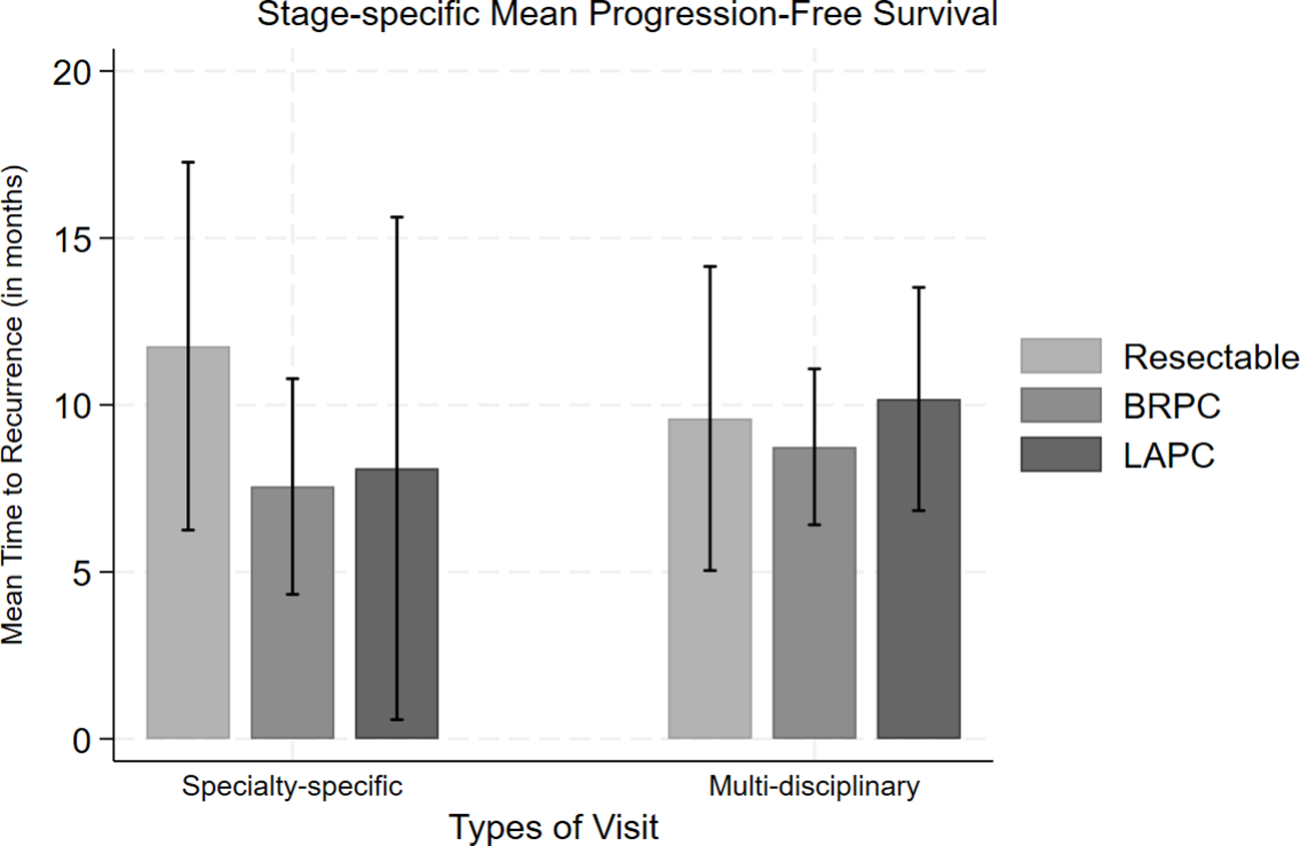
Comparison of mean progression-free survival along with standard error bars for each stage (resectable, borderline resectable (BRPC) and locally advanced (LAPC)) of pancreatic cancer based on their type of clinic visit.

The variation in treatment characteristics between patients seen in PMDC versus patients seen only through individual specialty clinics is summarized in **Table 2**. Patients in the PMDC group received an additional month of chemotherapy (median: 4; IQR: 3-5 months) compared to patients in the individual specialty clinic group (median: 3; IQR: 2-4 months) (p=0.028). However, when stratified by stage, the differences in median duration of chemotherapy were observed only in patients who had borderline resectable pancreatic cancer (Median [IQR] 4.0 (3.0, 5.0) vs Median [IQR] 3.0 (3.0, 4.0); p=0.021) (**Figure 1**). Variation was also seen in the type of first chemotherapy administered. A higher proportion of patients were started on a 5-FU based chemotherapy regimen in the PMDC cohort compared to in the individual specialty clinic cohort (n=216, 83.7% vs n=106, 72.1%), whereas a higher proportion of patients were started on a gem-based chemotherapy regimen in the individual specialty clinic group (n=38, 25.8% vs n=40, 15.5%). This variation in initial chemotherapy was statistically significant (overall p=0.018).

**Table 2:** Distribution of treatment characteristics based on stages of disease.

	Individual Specialty Clinic (n=149, 34.8%)	PMDC (n=267, 64.2%)	p-value
Overall			
Total duration of Chemotherapy, Median (IQR)	3.0 (2.0, 4.0)	4.0 (3.0, 5.0)	0.028
Administration of Radiation	41 (27.5%)	144 (53.9%)	<0.001
Neoadjuvant Completion	44 (48.9%)	124 (69.3%)	0.001
Clinical Trial enrollment	13 (8.7%)	46 (17.6%)	0.014
**Resectable**	59 (39.6%)	88 (33.0%)	
Total duration of Chemotherapy	3.0 (2.0, 4.0)	3.0 (2.0, 4.0)	0.201
Administration of Radiation	5 (8.5%)	10 (11.4%)	0.571
Neoadjuvant Completion	–	–	
Clinical Trial enrollment	6 (10.2%)	12 (13.6%)	0.530
**Borderline**	63 (42.3%)	90 (33.7%)	
Total duration of Chemotherapy	3.0 (3.0, 4.0)	4.0 (3.0, 5.0)	0.021
Administration of Radiation	19 (30.2%)	56 (62.2%)	<0.001
Neoadjuvant Completion	31 (49.2%)	59 (65.6%)	0.043
Clinical Trial enrollment	5 (7.9%)	10 (11.4%)	0.488
**Locally advanced**	27 (18.1%)	89 (33.3%)	
Total duration of Chemotherapy	4.0 (3.0, 6.0)	4.0 (3.5, 6.0)	0.543
Administration of Radiation	17 (62.9%)	78 (87.6%)	0.004
Neoadjuvant Completion	13 (48.1%)	65 (73.0%)	0.016
Clinical Trial enrollment	2 (7.4%)	24 (28.2%)	0.026

IQR, Interquartile range; Neoadjuvant completion is defined as completion of total recommended neoadjuvant therapy which is at least 4 months of chemotherapy with and without radiation for borderline and 4-6 months of chemotherapy with radiation for locally advanced pancreatic cancer per institutional standards. Please note that resectable PC was not included in the neoadjuvant completion calculation per institutional standards. PMDC: Pancreas Multidisciplinary Clinic.

Similarly, more than half of patients in the PMDC group received neoadjuvant radiation (n=144, 53.9%) while only 27.5% (n=41) of patients in the individual specialty clinic group received neoadjuvant radiation (p=0.001), with this difference driven by the borderline resectable (n=56, 74.7%; vs n=19, 25.3%; p<0.001) and locally advanced (n=78, 87.6% vs n=17, 62.9%; p=0.004) populations. In the PMDC group, most of the patients received SBRT (n=114, 79.2%) compared to conventionally fractionated radiation (n=30, 20.8%), while in the specialty specific clinic group, a similar number of patients received SBRT (n=22, 53.6%) and conventionally fractionated radiation (n=19, 46.3%) (overall p=0.005).

Among patients with borderline and locally advanced PDAC, 62.4% (n=169) of the patients completed recommended NAT per institutional standards. Those patients seen in PMDC were more likely to complete NAT per institutional standards as compared to patients who were seen only in specialty specific clinics (n=124, 69.3% vs n=44, 48.9%; p=0.001). This difference held true within both the borderline resectable (BRPC: n=59, 65.5% vs n=31, 49.2%; p=0.043) and locally advanced populations (LAPC: n=65, 73.0% vs n=13, 48.1%; p=0.016). Moreover, clinical trial enrollment was significantly higher in the PMDC group compared to individual specialty clinic group (n=46, 17.6% vs n=13, 8.7%; p=0.014), which was primarily driven by differences in the LAPC population (n=24, 28.2% vs n=2, 7.4%; p=0.026). In addition, 88.7% (n=235) of the patients in the PMDC group underwent genetic testing prior to surgery, compared to 61.5% (n=91) of patients seen only in individual specialty clinic (p<0.001).

### 3.3 Oncologic and pathologic outcomes

With respect to pathologic outcomes at the time of surgery, pathologic treatment response in the PMDC group was much higher compared to the individual specialty clinic group (p=0.004) (**Table 3**). Several other significant secondary outcomes are detailed in **Table 3**. Among those who had follow-up data, about half of the patients had recurrence (n=173, 47.9%). The median PFS was 8 (IQR: 4-12) months in the PMDC group compared to 5 (IQR: 2-15) months in the individual specialty clinic group (p=0.309). **Figure 1** shows differences in mean PFS based on stage and the type of clinic. A total of 86 (n=20.7%) patients died, and the median OS was not different in the PMDC group and individual specialty clinics group (median [IQR]: 9 [5-17] vs 9 [5-16] months; p=0.757).

**Table 3:** Primary and secondary outcomes for patients receiving neoadjuvant therapy.

	Total	Individual Specialty Clinic (n=149, 34.8%)	PMDC (n=267, 64.2%)	p-value
Genetic Testing	326 (78.9%)	91 (61.5%)	235 (88.7%)	<0.001
Pathologic Outcomes				
Treatment Response				0.004
No to poor Response	100 (24.1%)	50 (33.8%)	50 (18.7%)	
Moderate Response	248 (59.8%)	81 (54.7%)	167 (62.5%)	
Marked Response	55 (13.2%)	15 (10.1%)	40 (15.0%)	
Pathologically Complete Response	12 (2.9%)	2 (1.3%)	10 (3.7%)	
Lymph Node involvement	231 (55.5%)	99 (66.4%)	132 (49.4%)	0.001
Positive Surgical Margins	55 (13.2%)	22 (14.8%)	33 (12.4%)	0.487
Oncologic Outcomes				
Progression-free Survival, Median (IQR) in months	8 (4-12)	5 (2-15)	8 (4-12)	0.309
Overall Survival, Median (IQR) in months	9 (5-16)	9 (5-17)	9 (5-16)	0.757

IQR, Interquartile range. PMDC: Pancreas Multidisciplinary Clinic.

### 3.4 Association between clinic type and outcomes

**Figure 2:**
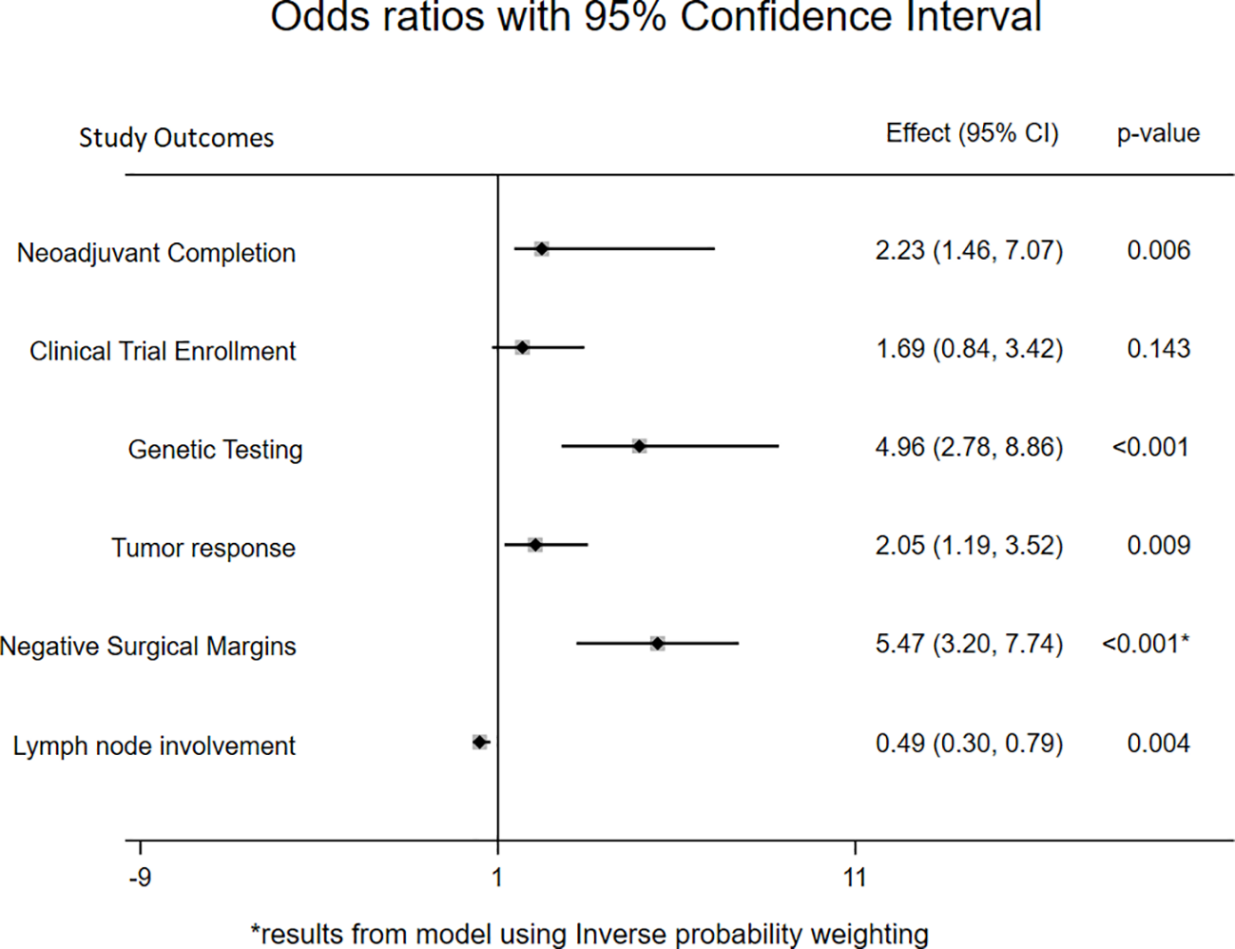
Forest plot showing odds ratio with 95% confidence interval for primary and secondary outcomes comparing PMDC and individual specialty clinic. *represents that the regression model for negative surgical margins used inverse probability weighting.

On multivariable level, the odds of completing NAT per institutional standards was 2.23 times more likely in the PMDC group compared to the specialty specific clinic group (OR:2.23, 95CI: 1.46-7.07; p=0.006) (**Figure 2**). In addition, with every additional PMDC visit, the odds of completion of recommended NAT increased by 1.88 times compared to the patients who went to PMDC only once (OR: 1.88; 95CI: 1.05-3.35, p=0.34). Interestingly, while clinical trial enrollment was significantly higher among the PMDC group on univariable analysis (OR:2.24 95CI: 1.17-4.30; p=0.015), there was no significant statistical difference between the groups after controlling for covariates (OR: 1.69, 95CI: 0.84-3.42; p=0.143). In contrast, the odds of receiving either germline or somatic testing prior to surgery was higher in patients seen through PMDC compared to those seen in individual specialty clinics after controlling for covariates (OR: 5.66, 95CI: 3.05-10.50; p<0.001). Of note, patients in the PMDC group had twice the odds of getting moderate to complete neoadjuvant response compared to the individual specialty clinic group (OR:2.05, 95CI: 1.19-3.52; p=0.009). Similarly, patients in the PMDC group had 0.49 times lower odds of positive lymph node involvement at the time of resection compared to the individual specialty clinic group (OR: 0.49, 95 CI: 0.30-0.79; p=0.004). Despite statistically significant higher odds of neoadjuvant completion per institution standards and increased pathologic tumor response in PMDC group, on univariable analysis, no association between R0 resection and clinic type was initially observed. However, after adjusting for covariates (age, sex, race, stage, baseline CA 19-9 level, total duration of chemotherapy, and neoadjuvant radiation) using propensity score matching, the odds of an R0 section were about 5 times higher in the PMDC group compared to the individual specialty clinic group (OR:5.47, 95CI: 3.20-7.74; p<0.001).

On using inverse-probability weights to estimate treatment effects, the average effect of attending PMDC on PFS was 4.45 (95CI: 0.87-8.04; p=0.015) months. Particularly, the average PFS would be 11.19 (95CI: 11.10-19.23) months if all patients were seen in PMDC as opposed to 6.74 (95CI: 3.97-9.52; p<0.001) months if all patients were seen in only an individual specialty clinic. No significant difference in average treatment effect of PMDC visits was observed on OS (1.26 95CI:-2.44 to 4.56; p=0.477).

## 4 Discussion

Given that management of pancreatic cancer requires multi-disciplinary care, the use of pancreatic cancer multidisciplinary clinics has been increasing. In the present study, our main goal was to examine whether evaluation at a PMDC versus individual specialty clinics at the same institution had an impact on adherence to the treatment recommendations based on the institutional standards. Indeed, patients who were evaluated at our PMDC at any point during their pre-operative care were twice as likely to complete the stage-specific recommended NAT per institutional standards compared to patients seen only in individual specialty clinics. Perhaps as a result, patients in the PMDC group had better tumor response to NAT and underwent more R0 resections. Similarly, patients seen in PMDC were more likely to receive ancillary services such as genetic testing prior to surgery compared to patients who were seen in individual specialty clinics. Of importance, there was a significant association between longer PFS and visits through the PMDC. While there was no difference in OS to-date, the study period was 2019-2022, and as such, further follow-up will be needed to understand if an OS benefit eventually be realized.

Commonly, an MDC includes providers such as a medical, surgical and radiation oncologist, radiologist, social worker, genetic counselor and dietitian (17). Previous studies have demonstrated the effectiveness of a one-day MDC in standardization of treatment decisions, reduced patient visits, and improved patient satisfaction (2, 12, 14). In addition, previous study by our group has demonstrated the feasibility of using tools such as a malnutrition assessment early in the disease course through the PMDC (18). Despite the recognition of the benefits of MDCs, its value in provision of institution based guideline-concordant care as compared to individual specialty specific oncology clinics had been previously unknown. As such, the current study is important in that it adds to the existing literature by providing insights about differences in practices based on clinic type and its impact on quality of care and patients’ oncologic outcomes.

In addition to the efforts towards establishing evidence-based standardization of care for PDAC, the NCCN recommends the utilization of ancillary services such as genetic counseling, nutritional support, and palliative care to improve patients’ quality of life and optimize treatment decisions (19). Importantly, the current study examined the association between PMDC and genetic testing prior to surgery. Pishvaian et al. found actionable genetic alterations in over 25% of the pancreatic cancer patients with significantly increased progression free survival among the patients who received matched therapies (20). However, access to genetic testing remains poor in the PDAC population (21). MDC potentially provides an ideal setting to integrate genetic education and testing for identification of actionable mutations. Everett et al. reported significantly improved compliance for genetic testing among PDAC patients due to reduction in logistical access barriers after incorporating genetic testing into the MDC (22). In addition, incorporation of genetic testing and counseling during PMDC enables maximization of utility of testing resources, improves team communication and reduces the burden of physicians to make referrals (22, 23) Of interest, while we did not have information on patients who received matched therapies, the current study found about 4 months longer time to recurrence among patients in the PMDC group as compared to patients in the individual specialty clinic group.

In addition, the current study found that compared to the specialty specific clinic group, a higher proportion of patients who identified as white had a PMDC visit. The pancreatic cancer treatment through PMDC has shown to mitigate the socioeconomic disparities related to treatment and overall survival (13). Furthermore, MDC can have higher health system utilization due to involvement of various specialists as noted by policy experts which raises the concern of increased health care cost (17). Conversely, MDCs for lung cancer have shown to reduce patient visits and led to both patient and health system cost savings (24). Also, patients evaluated at a lung cancer MDC had lower emergency department visits during their treatment compared to those who did not participate in the MDC (25).

Given these benefits that MDC care can provide, efforts to more broadly reorganize cancer care to deliver inter-disciplinary coordinated care to pancreatic cancer patients should be incentivized and pursued (26). Given the resources required to support MDCs, additional efforts to characterize the cost-effectiveness of providing such inter-disciplinary care will be important (11, 27).

### 4.1 Limitations

The current study had limitations that need to be considered when interpreting the results. The retrospective design of the current study may have led to selection bias. In particular, our study population was restricted to resectable, borderline, and locally advanced diseases who eventually received surgical resection. This decision was based on available institutional data, and there may have been associations with the ability to undergo surgery that affected the relationship between initial clinic visit type and oncologic outcomes. Certainly, future analysis that examined the impact of initial clinic visit type in all patients with newly diagnosed, non-metastatic patients would be of great interest. Furthermore, the PDAC patient population often undergoes consultation from more than one center and may have presented to our or other centers initially for a second opinion. As such, patients grouped in the individual specialty clinic group for the study could have undergone MDC evaluation at other centers leading to misclassification bias. In addition, due to lack of granular details on the follow-up practice patterns in our institutional database, variation in the follow-up between clinics and its potential impact on survival outcomes could not be accounted in the analysis. While all patients received their surgical resection in the JHH, many received NAT locally at an outside institution, which may have influenced outcomes. Importantly, ECOG status during the first visit was unknown for 24.8% among the individual specialty clinic cohort compared to 3.0% among PMDC cohort. Consequently, there is a possibility that more patients seen only in individual specialty clinics had lower performance status which led the providers to choose gemcitabine-based neoadjuvant agent more frequently. In addition, our institutional database doesn’t have the data to calculate the proportion of patients who started treatment prior to JHH evaluation. However, multivariable analyses were performed to adjust for ECOG and other covariates. Furthermore, our study was a single-institutional retrospective cohort study, therefore, the generalizability of the findings should be done with caution.

### 4.2 Conclusion

In summary, patients who participated in PMDC were twice as likely to complete recommended NAT per institutional standards as compared to patients who were seen only through specialty specific clinics. While our analysis did not show a difference in OS to-date among surgically resected patients, those who participated in PMDC experienced better tumor response to NAT, a higher rate of R0 resections, and longer PFS. In addition, genetic testing prior to surgery was more frequently completed among patients who had PMDC visit. Future efforts to incentivize the transition from traditional siloed oncology visits to MDC care are warranted.

## Data availability statement

The data analyzed in this study is subject to the following licenses/restrictions: we are not allowed to move the data from the safe desktop authorized by the Johns Hopkins IRB. Requests to access these datasets should be directed to PP, ppathak7@jhmi.edu.

## Ethics statement

Ethical approval was not required for the study involving humans in accordance with the local legislation and institutional requirements. Written informed consent to participate in this study was not required from the participants or the participants’ legal guardians/next of kin in accordance with the national legislation and the institutional requirements.

## Author contributions

PP: Conceptualization, Data curation, Formal Analysis, Investigation, Methodology, Project administration, Resources, Software, Validation, Visualization, Writing – original draft, Writing – review & editing. AH-P: Writing – review & editing. JMH: Writing – review & editing. LZ: Writing – review & editing. JH: Writing – review & editing. AN: Writing – review & editing, Conceptualization, Funding acquisition, Project administration, Resources, Supervision, Validation, Visualization, Writing – original draft.
