# Supplementary material for: Variation in outcomes and practice patterns among patients with localized pancreatic cancer: the impact of the pancreatic cancer multidisciplinary clinic
Source: Front Oncol. 2024 Jul 11;14:1427775. doi: 10.3389/fonc.2024.1427775 (PMC11269111; doi:10.3389/fonc.2024.1427775)
Supplement: Supplementary file 1 [file DataSheet_1.docx]

**Supplement 1**

Pathologic end points of interest: Using the College of American Pathologists' Protocol for the Examination of Specimens From Patients With Carcinoma of the Exocrine Pancreas tumor recommended grading system (17), we categorized the neoadjuvant tumor response as: grade 0, complete response (no viable cancer cells); grade 1, moderate response (single cells or small groups of cancer cells); grade 2, minimal response (residual cancer outgrown by fibrosis); and grade 3, poor response (minimal or no tumor kill; extensive residual cancer) (18). For multivariable analysis in the study, moderate to complete response was categorized as a *good* tumor response, whereas minimal or no response was categorized as a *poor* tumor response.

Lymph node status was categorized as *positive* if any of the harvested nodes during resection were positive for malignancy and *as negative* if malignancy was not found in any of the harvested lymph nodes during resection.

For surgical margin status, resection was classified as R0 if there was no microscopic tumor present at any of the margins (uncinate, pancreatic neck, or bile duct), R1 if microscopic residual tumor was present at any margin, and R2 if gross residual disease was remaining after completion of surgical dissection (3). We defined *negative* surgical margins as a R0 resection, while R1 and R2 resections were categorized as *positive* surgical margins.

Table 4: Odds ratios for primary and secondary outcomes comparing Multidisciplinary Clinic visit versus Standard Oncology Clinic visit.

|  | **Reference** | **cOR (95 CIs)** | **p-value** | **aOR (95CIs)** | **p-value** |
| --- | --- | --- | --- | --- | --- |
| Neoadjuvant Completion | No | 2.35 (1.40-3.97) | 0.001 | 2.23 (1.46-7.07) | 0.006 |
| Clinical Trial Enrollment | No | 2.24 (1.17-4.30) | 0.015 | 1.69 (0.84-3.42) | 0.143 |
| Genetic Testing before Surgery | No | 4.90 (2.96-8.12) | <0.001 | 4.96 (2.78-8.86) | <0.001 |
| Moderate to complete Neoadjuvant response | Poor to no response | 2.21 (1.40-3.50) | 0.001 | 2.05 (1.19-3.52) | 0.009 |
| Negative Surgical Margins | Positive | 1.23 (0.69-2.20) | 0.488 | 5.47 (3.20-7.74) | <0.001* |
| Lymph node involvement | No involvement | 0.49 (0.32-0.75) | 0.001 | 0.49 (0.30-0.79) | 0.004 |
|  |  |  |  |  |  |

*cOR: Crude Odds Ratio (Univariable analysis); aOR: Adjusted Odds Ratio (Multivariable analysis). Based on clinical relevance, each multivariable regression model was controlled for age, sex, race, performance status, stage, neoadjuvant therapy, and duration of chemotherapy. *Inverse proprability weighting was done to find association between and surgical margin with clinic types as treatment groups, controlling for total duration of chemotherapy, neoadjuvant radiation, age, sex, race and stage, treatment naïve CA 19 9 and treatment response.*

**References**:

1. Washington K, Berlin J, Branton P, Burgart LJ, Carter DK, Crompton CC, et al. Protocol for the Examination of Specimens From Patients With Carcinoma of the Pancreas. Pancreas (Exocrine) Jan 2016; 3.3.0.1

2. Hartman DJ, Krasinskas AM. Assessing Treatment Effect in Pancreatic Cancer. Arch Pathol Lab Med. 2012 Jan 1;136(1):100–9.

3. Wittekind C, Compton C, Quirke P, Nagtegaal I, Merkel S, Hermanek P, et al. A uniform residual tumor (R) classification. Cancer. 2009;115(15):3483–8.
